# Crosstalk between the CBM complex/NF-κB and MAPK/P27 signaling pathways of regulatory T cells contributes to the tumor microenvironment

**DOI:** 10.3389/fcell.2022.911811

**Published:** 2022-07-19

**Authors:** Tongbing Qi, Ying Luo, Weitong Cui, Yue Zhou, Xuan Ma, Dongming Wang, Xuewen Tian, Qinglu Wang

**Affiliations:** ^1^ College of Sport and Health, Shandong Sport University, Jinan, China; ^2^ Key Laboratory of Biomedical Engineering and Technology of Shandong High School, Qilu Medical University, Zibo, China; ^3^ Clinical Laboratory, Zibo Central Hospital, Zibo, China; ^4^ Department of Pediatrics, People’s Hospital of Huantai, Zibo, China

**Keywords:** regulatory T cells, tumor microenvironment, CBM signaling pathway, MAPK/P27 signaling pathway, CARMA1

## Abstract

Regulatory T cells (Tregs), which execute their immunosuppressive functions by multiple mechanisms, have been verified to contribute to the tumor microenvironment (TME). Numerous studies have shown that the activation of the CBM complex/NF-κB signaling pathway results in the expression of hypoxia-inducible factor-1 (HIF-1α) and interleukin-6 (IL-6), which initiate the TME formation. HIF-1α and IL-6 promote regulatory T cells (Tregs) proliferation and migration through the MAPK/CDK4/6/Rb and STAT3/SIAH2/P27 signaling pathways, respectively. IL-6 also promotes the production of HIF-1α and enhances the self-regulation of Tregs in the process of tumor microenvironment (TME) formation. In this review, we discuss how the crosstalk between the CARMA1–BCL10–MALT1 signalosome complex (CBM complex)/NF-κB and MAPK/P27 signaling pathways contributes to the formation of the TME, which may provide evidence for potential therapeutic targets in the treatment of solid tumors.

## Highlights

1) The activation of the CBM complex/NF-κB signaling pathway results in TME formation.
2) HIF-1α and IL-6 promote Treg proliferation and migration via the MAPK/CDK4/6/Rb and STAT3/SIAH2/P27 signaling pathways.
3) The crosstalk between the CBM complex/NF-κB and MAPK/P27 signaling pathways appears in Tregs.

## Introduction

The development of immune checkpoint therapy (ICT), which stimulates an immune response to cancer, has been one of the most rapid and important advances in cancer treatment over the past decade. Programmed cell death 1 (PD-1) is an immunosuppressive co-stimulatory signal receptor that belongs to the CD28 family. PD-1 and PD-L1 blockage at immune checkpoints can rejuvenate patients’ T cells to achieve long-term remission (Qi et al., 2020). However, the clinical effect of programmed cell death 1(PD-1)/programmed cell death 1 ligand 1 (PD-L1) targeted therapy for solid malignant tumors is not ideal (Cai et al., 2019). Only 20% of patients achieve favorable long-term results after treatment, and most patients relapse after treatment (Yan et al., 2019). This phenomenon may be related to the tumor microenvironment (TME), which is characterized by nutrient competition, hypoxia, low pH, and metabolite accumulation. Such complex conditions accelerate exhaustion of T effector cells and promote differentiation and accumulation of regulatory T cells (Tregs), M2-like macrophages, and Myeloid-derived suppressor cells (MDSCs). The TME also produces unique subsets of myeloid cells known as tumor-associated dendritic cells (TADC) and tumor-associated neutrophils (TAN) (Bader et al., 2020; Wang et al., 2021).

In this complex microenvironment, T cells encounter many inhibitory cells and molecules that can disrupt the survival, activation, proliferation, and effector functions of T cells (Joyce and Fearon, 2015; Turley et al., 2015). Alongside the developments in antibody therapy, modulation of cell signaling pathways using small-molecule inhibitors has gained ground within the immunotherapy field. The functional profiles of immune cells are necessarily shaped in response to environmental cues, which are conveyed to the cellular machinery *via* a myriad of distinct but overlapping signaling cascades (Wicherska-Pawlowska et al., 2021).

Recent studies have shown that Tregs may be involved in PD-1/PD-L1 blockage treatment, and the PD-1/PD-L1 axis may affect the differentiation and function of Tregs (Cai et al., 2019). Tregs execute their immunosuppressive functions by multiple mechanisms, such as by consuming interleukin-2 (IL-2), expressing cytotoxic T-lymphocyte-associated protein 4 (CTLA-4), secreting inhibitory cytokines (transforming growth factor-β, interleukin-10, interleukin-35) (Takeuchi and Nishikawa, 2016), and directly killing T cells or Antigen-presenting cells (APCs) by producing granular enzymes and perforin (Sakaguchi et al., 2010; Cai et al., 2019). These functions can be enhanced by classical interleukin-6 (IL-6) receptor (IL-6R) signaling (Hagenstein et al., 2019). These functions of Tregs may be related to hypoxia in the TME. The microenvironment of most tumors is usually hypoxic, and the expression level of hypoxia-inducible factor-1α (HIF-1α) is often increased in Treg (He et al., 2015). Hypoxia can also change the T cells CBM complex (CARMA1-BCL10-MALT1) activity, which is closely related to the development of solid tumor via NF-κB activation (Schaefer, 2020).

The effects of the hypoxic environment on the immunosuppressive function of Tregs are still inconclusive. Some studies have shown that HIF-1α positively affects the function of Tregs and plays a role in their suppressive function in tumors (Clambey et al., 2012; Westendorf et al., 2017). Other studies, however, have shown the opposite (Hsu and Lai, 2018). A recent study using a murine model of glioma has shown that ablation of HIF-1α leads to enhanced animal survival due to a decrease in the migratory abilities of HIF-1α Knockout Tregs (Miska et al., 2019). Here, we provide a brief review of signaling pathways in Tregs and the formation of the TME (Table 1).

**TABLE 1:** Points of concern between TME, Treg, CDKs, HIF-1α, CBM, MAPK, SIAH2 and STAT.

Correlation	Points of concern	References
TME and Treg	Treg is one of the important factors in the formation of tumor microenvironment	Sakaguchi et al. (2010)
CDKs and TME	TME was changed by drug targeting CDKs	Johnson et al. (2010)
HIF-1α and TME	Treg HIF-1α expression in TME is increased	He et al. (2015)
	SIAH2/PHD/HIF-1α pathway plays key role in the development of the TME	Nakayama et al. (2004)
		Albadari et al. (2019)
HIF-1α and Treg	Treg HIF-1α expression in TME is increased	He et al. (2015)
	HIF-1α positively affects Treg function	Westendorf et al. (2017)
	VEGF and VEGF receptor are closely related to HIF-1α in Tregs	Vaupel and Multhoff, (2018)
CBM complex and Treg	Partial disruption of CBM complex in Tregs improve immune checkpoint therapy	Di Pilato et al. (2019)
MAPK and Treg	MAPK regulates the Treg cell cycle and gene expression	Klomp et al. (2021)
	ERKs adjust Treg proliferation, differentiation, et al	Wang et al. (2019)
STAT and Treg	IL-6/STAT1/3 promote Treg proliferation	Yeh et al. (2013)
SIAH2 and Treg	immunosuppressive function of Siah2^−/−^ Tregs was blunted	Nakayama et al. (2004)
CDKs and Treg	CDKs regulate Treg growth, proliferation, dormancy, and apoptosis	Arnett et al. (2021)
	CDK4/6 phosphorylation affects Tregs proliferation and migration	Scortegagna et al. (2020)

## Cyclin-dependent kinase signaling enhances treg proliferation and migration

**FIGURE 1:**
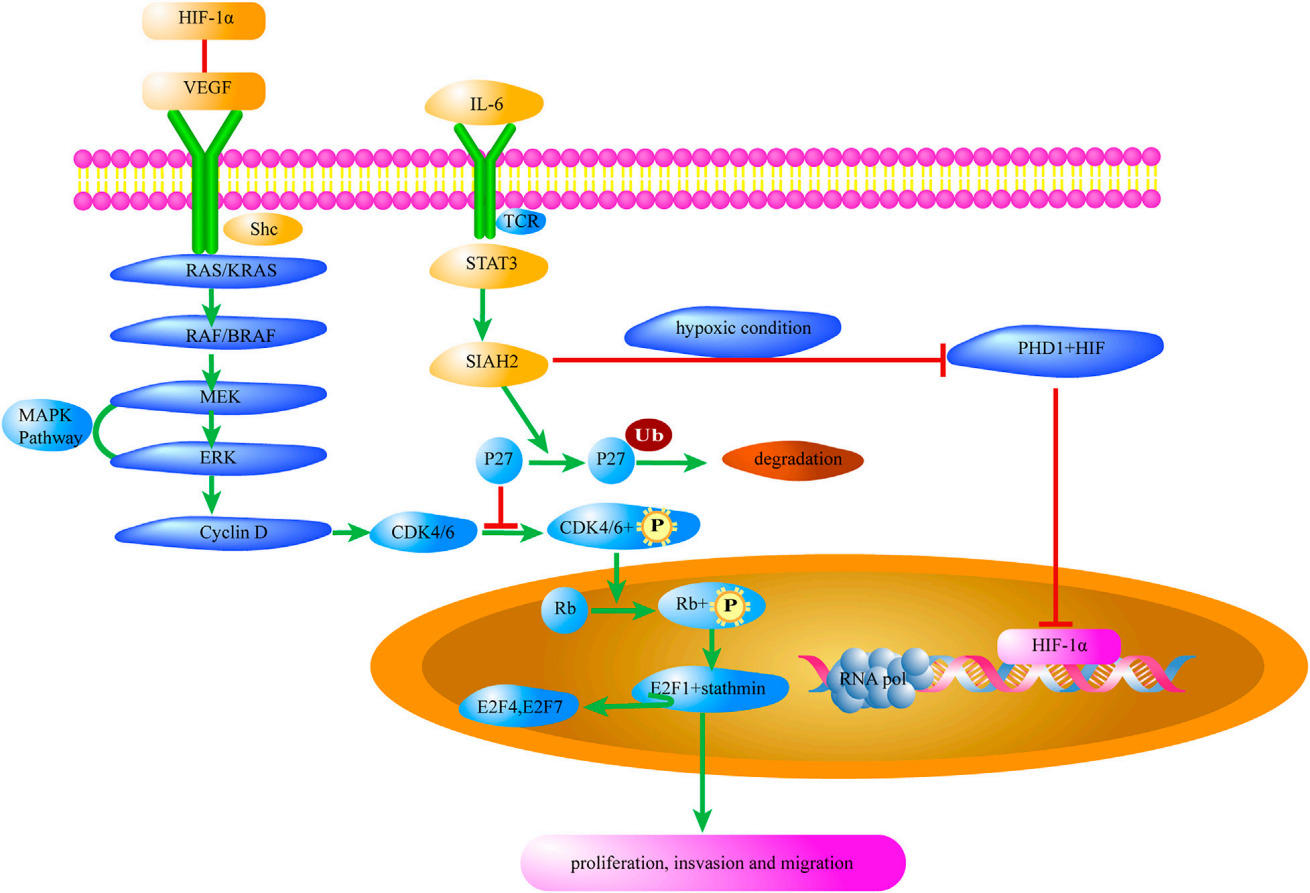
MAPK/P27 signaling of Tregs contributes to the tumor microenvironment. Il-6 in the tumor microenvironment promotes the expression of HIF-1α in Tregs and removes the inhibition of CDK4/6 by P27. Meanwhile, the interaction between HIF-1α and VEGF activates the MAPK/CDK4/6 signaling pathway to promote Treg cell proliferation. HIF-1α, Hypoxia-Inducible Factor-1α; VEGF, Vascular endothelial growth factor; shc, Src-homology collagen protein; RAS, small G-protein; KRAS, V-Ki-ras2 Kirsten ratsarcoma viral oncogene homolog; RAF, Raf kinase; BRAF, v-raf murine viral oncogene homolog B1; MEK, Mitogen-activated protein kinase; ERK, Extracellular-signal-regulated kinase; MAPK, mitogen-activated protein kinase; CDK, Cyclin-dependent kinase; Rb, Retinoblastoma protein; E2F, Transcription factor; P27, Potential tumor suppressor protein; Ub, Ubiquitin; SIAH2, Seven in absentia homolog 2; STAT3, Signal Transducer and Activator of Transcription 3; TCR, T cell receptor; PHD1, Hypoxia-inducible factor prolyl hydroxylase 1.

Cyclin-dependent kinases (CDKs) are a class of serine/threonine kinases. As important signaling molecules that regulate transcription, CDK–cyclin complexes are involved in Treg growth, proliferation, dormancy, and apoptosis (Arnett et al., 2021). During the Treg cycle, cyclins are expressed and degraded periodically and are bound to the CDK activated by them. Through the activity of CDKs, phosphorylation of different substrates can be catalyzed to realize the promotion and transformation of the Treg cycle. Sequential phosphorylation of CDK4/6 and Rb proteins activates 1) downstream E2F and Stathmin, leading to the release of transcription factors such as E2F4 and E2F7 (Dyson, 1998; Pietrzak et al., 2018), and 2) some genes necessary for E2F4 and E2F7 activation and transcription, leading to progression into the S phase (Geng et al., 2020). As shown in Figure 1, when CDK4/6 phosphorylation is inhibited by P27 which is a CDK inhibitor (Scortegagna et al., 2020), some functions of Tregs such as gene transcription, cell proliferation, and migration, are affected.

Therefore, it is a good strategy to control the TME by inhibiting Treg proliferation by targeting CDKs with some target drugs (Johnson et al., 2010). Numerous clinical trials have been conducted with small molecules that target CDKs in patients with cancer (Albanese et al., 2010).

## IL-6 enhances effects of the STAT3/SIAH2/CDK4/6 signaling pathway

The IL-6 signaling pathway is associated with tumor angiogenesis, and previous studies have found that the suppression of IL-6 signaling led to suppression of angiogenesis and migration of breast cancer (Luo et al., 2020). IL-6 is produced by fibroblasts, keratinocytes, and endothelial cells in response to injury, and corresponding receptors also exist on Tregs (Hagenstein et al., 2019). IL-6 transmits signals resulting in the activation of transcription factors, signal transducers and activators of transcription 1 (STAT1) and 3 (STAT3), through the association with gp130, and then promotes Foxp3 (+) Treg proliferation (Yeh et al., 2013).

STAT3 is an important member of the STAT family, which also includes STAT1, STAT2, STAT4, STAT5a, and STAT5b. The STAT6 and STAT3 signal transduction pathways are closely related to cell proliferation, differentiation, and apoptosis. The pathways control the production of growth factors and cytokines, and the extracellular signal stimulation, thereby regulating target gene transcription. The pathway activation can lead to abnormal cell proliferation and malignant transformation (Buettner et al., 2002). Jak–STAT3 can be activated by a variety of extracellular proteins, such as interleukins (Wang and Fuller, 1994). When activated, JAKs phosphorylate the tyrosine site on the receptor, causing the receptor to produce a region that binds to STAT3. At this point, the Src homology (SH2) domain on STAT3 binds to the phosphorylated tyrosine residues on the receptor, thereby forming homo- or heterodimers, which are transported to the nucleus and interact with other transduction factors to regulate gene transcription (Liu et al., 2017).

STAT3 activates the transcription and translation of the ubiquitin ligase, Seven In Absentia Homolog 2 (SIAH2). Then, P27, which inhibits CDK4/6 activation, is degraded by ubiquitination of SIAH2 (Figure 1). According to Hoshino et al., upregulation of P27 expression is necessary for specific blockage of tumor extracellular signal-regulated kinase pathways, which in turn leads to complete growth inhibition of tumor cells (Hoshino et al., 2001). A recent study has shown that the immunosuppressive function of Tregs in tumors of *Siah2* ^−/−^ mice was blunted owing to P27-dependent suppression of CDK4/6 signaling activation (Scortegagna et al., 2020).

STAT3 gene is highly expressed in hepatocellular carcinoma cells, and regorafenib (Stivarga), a drug targeting STAT2 for the treatment of hepatocellular carcinoma, has been identified as a second-line oral agent (Jindal et al., 2019).

## SIAH2 enhances tumor HIF-1α expression

The SIAH2/PHD/HIF-1α pathway plays an important role in the development of the TME. The experiments by Nakayama et al. have confirmed that the E3 ubiquitin ligase SIAH2 shows significant ubiquitin-dependent degradation of prolyl-hydroxylase 1 (PHD1) and prolyl-hydroxylase 3 (PHD3), while its effect on PHD2 is not significant (Nakayama et al., 2004). There are two types of HIF-α, including HIF-1α and HIF-2α(Albadari et al., 2019). Among them, only the expression mechanism of HIF-1α has been well studied, and only HIF-1α has been found in a wide range of cells. As a substrate for PHD, HIF-1α can be hydroxylated in two forms, thereby undermining the stability of HIF-1α. When the intracellular oxygen concentration is below normal values (2–5%), a hypoxic environment is generated. Induction of SIAH2 expression by hypoxia serves to enhance the degradation of prolyl-hydroxylase 1/3(PHD1/3) and consequently increase the abundance of HIF-1α(Nakayama et al., 2004).

Upregulated HIF-1α expression in tumor cells and immune cells is characteristic of the TME. Some studies have shown that HIF-1α positively affects Treg function and plays a role in their suppressive function in tumors (Clambey et al., 2012; Westendorf et al., 2017). An immune escape mechanism involves Treg-mediated immunosuppression, which is used by tumors to overcome the antitumor activity of CD8^+^ cytotoxic T cells, dendritic cells, and natural killer cells (Bader, Voss, and Rathmell, 2020).

When the expression level of HIF-1α in Tregs is upregulated, hypoxia response element (HRE) binds to HIF-1α, resulting in the production of a variety of products, such as vascular endothelial growth factor (VEGF), which is associated with angiogenesis, and CXCR4, which is associated with cell migration (Forsythe et al., 1996). Overexpression of VEGF and activation of VEGF receptor are closely related to HIF-1α in Tregs (Vaupel and Multhoff, 2018).

VEGF and HIF-1α are also closely related to the development of blood vessels. Currently, inhibitor drugs targeting these two proteins are approved by the FDA for the treatment of some tumors (Jindal, Thadi, and Shailubhai, 2019). Given that VEGF and HIF-1α genes are also implicated in Treg reproduction, immunosuppressive drugs can be used to destroy the TME in Tregs.

## Mitogen-activated protein kinase signaling enhances CDK4/6 activation

The mitogen-activated protein kinase (MAPK) signaling pathway is a signal transduction pathway that is widely found in animal cells. This pathway plays an important role in regulating the Treg cell cycle and gene expression (Liu et al., 2013; Klomp et al., 2021). The MAPK signaling pathway consists of a cascade of successively activated serine/threonine protein kinases that amplify and transmit extracellular signals step by step to the cell and even to the nucleus, connecting membrane receptor-bound extracellular stimuli to effector molecules in the cytoplasm and nucleus (Tetu et al., 2021).

Vascular endothelial growth factors (VEGFs) constitute a subfamily of growth factors that stimulate the growth of new blood vessels. VEGFs are important signaling proteins involved in both vasculogenesis (*de novo* formation of the embryonic circulatory system) and angiogenesis (the growth of blood vessels from preexisting vasculature) (Negri et al., 2019). VEGFs can bind the Treg cell membrane receptor and increase Treg cell proliferation (Vasilev et al., 2019). NRP-1, a semaphorin III receptor involved in the activation of T cells, is constitutively expressed on the surface of Foxp3+ Tregs independently of their activation status. NRP-1 has been found to interact with VEGFs and interfere with Treg-mediated immunosuppression (Pucino et al., 2014).

RAS is a small GTP-binding protein, with the GTPase domain binding GDP in the inactive state and GTP in the active state; therefore, RAS plays the role of molecular switch. RAS adjusts T cell development, differentiation, and proliferation by inducing downstream signal transduction pathways. Inhibition of RAS has been found to be associated with an increased number and boosted function of Foxp3+ Tregs (Aizman et al., 2010).

ERKs adjust Treg proliferation, differentiation, and survival, and regulate the production of a variety of downstream growth factors (EGF, model NGF, and PDGF) (Liu et al., 2013; Wang et al., 2019). The RAS/Raf/MEK/ERK axis is the main axis of the ERK pathway (Akula et al., 2019). Activation of ERK can promote the phosphorylation of cytoplasmic target proteins or regulate the activity of other protein kinases; the activated ERK is translocated into the nucleus, where it promotes the phosphorylation of a variety of transcription factors. The MAPK pathway transfers extracellular stimulatory signals to cells and their nuclei to regulate cell growth, differentiation, proliferation, apoptosis, and migration.

As shown in Figure 1, VEGF-R2 phosphorylates and activates SHC, which binds to spline protein, which binds to guanylate exchange protein via the SH2 domain to approach the RAS, thereby further activating the MAPK cascade (Raf1→MEK1/2→ERK1/2). It can also induce the activation of P38 MAPK, which in turn activates MAPKK-2/3 and phosphorylates both the polymerized regulatory molecule of filamin actin (F-actin) and heat shock protein 27 (HSP27), causing the recombination of the actin cytoskeleton and stimulating endothelial cell migration (Wu et al., 2016).

## Effects of *CARMA1* on the NF-κB signaling pathway in tregs

**FIGURE 2:**
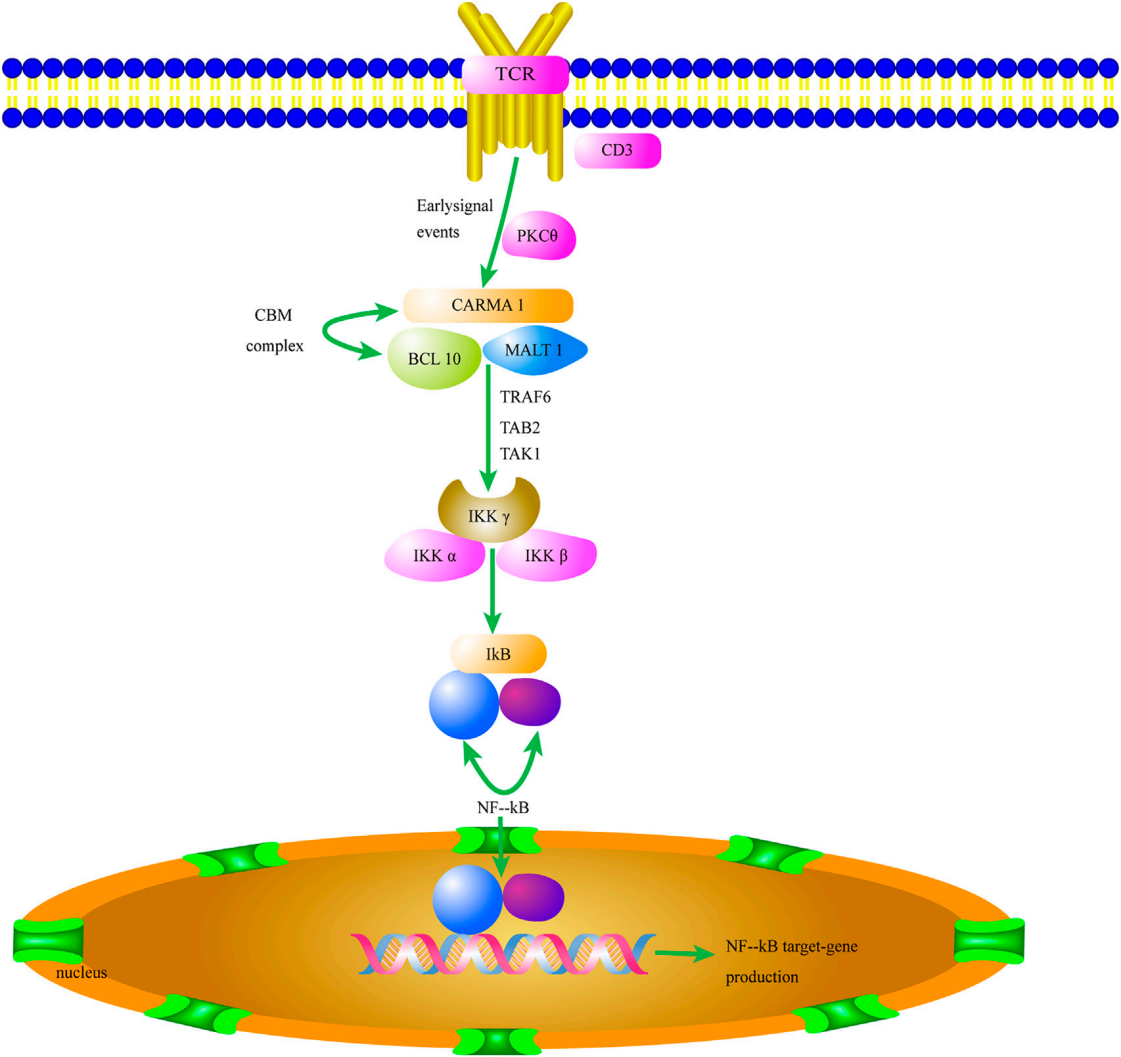
The CBM complex/NF-κB signaling pathway of Tregs contributes to the tumor microenvironment. Activation of the CBM complex/NF-κB signaling pathway in Tregs increases HIF-1α and IL-6 expression and initiates the TME. The secretion of HIF-1α and IL-6 in the TME promotes Treg proliferation and migration. TCR, T cell receptor; PKC, Protein kinase C; CARM1, Caspase recruitment domain-containing membrane-associated guanylate kinase protein-1; BCL10, B-cell lymphoma/leukemia 10; MALT 1, Mucosa-associated lymphoid tissue 1; TRAF6, TNF receptor associated factor 6; TAK1, TGFβ-activated kinase 1; TAB2, TAK1-binding protein 2; Iκκ, inhibitor of kappa kinase; NF-kB, nuclear factor kappa B.

CARMA1 (CARD11) proteins are composed of 1,147 amino acid residues. Their N-termini consist of a CARD domain and a coiled-coil structure, and the C-termini contain a PDZ domain, an SH3 domain, and a GUK domain. Activated downstream of protein kinase C (PKC), CARMA1 is coupled to lipid rafts on cell membranes (Figure 2). When MHC binds to molecules on the cell surface, TCR activates tyrosine phosphoric acid, which leads to activation of PKC. PKC is then phosphorylated and couples CARMA1 to the membrane, where BCL10 is connected to the Ig region of MALT1 and to CARMA1 to form a CBM complex. The CBM complex of all the T cells—which can respond to specific antigen receptor stimulation and includes the invariant components BCL10 and MALT1, assembled with CARD9, CARD10, CARD11, or CARD14—is an important mediator of NF-κB activation (Schaefer, 2020).

In addition, MALT1 interacts with CARMA1’s coiled spiral region. This complex has enzymatic activity (Oruganti et al., 2017). NF-κB usually forms homo-/heterodimers with P65 and P50 and is inactivated in the cytoplasm by binding to the inhibitory protein IκB to form a trimer complex. CARMA1 binds to lipid rafts on the cell membrane. It also acts as a signal transmitter and ultimately activates NF-κB when Tregs are stimulated by antigens (Di Pilato et al., 2019). CARMA1 plays an important role in the activation of the NF-κB signaling pathway as a junction between membrane-bound and cytoplasmic proteins (Ma et al., 2014).

The functional changes of the CBM complex in Tregs have an important relationship with the formation of TME. The CBM complex mediates TCR-induced NF-κB activation in Tregs and controls the conversion of resting Tregs to effector Tregs when needed. Partial disruption of the CBM complex in Tregs, such as due to a knockdown of *CARMA1* gene, can weaken the formed TME and improve sensitivity to PD-1/PD-L1 immune checkpoint therapy (Di Pilato et al., 2019). The interaction among CARMA1, BCL10, and MALT1 also affects the activity of downstream NK-KB signaling pathway, and the control of BCL10 on MALT1 paracaspase activity affects the formation of malignant melanoma TME (Rosenbaum et al., 2019). The role of Bcl10 in the development of Tregs and formation of TME is essential (Yang et al., 2021). Based on the results of a series of studies on the CBM complex, we can predict that drugs targeting CARMA1, BCL10 and MALT1 inhibitors will be able to effectively break the TME formed by Tregs (Keller et al., 2021).

The continuously activated NF-κB in Tregs or other cells can enhance the transcription of VEGF genes and also increase the levels of some tumor promoting cytokines, such as IL-1 (acute leukemia growth factor), TNF (malignant lymphogranuloma, T cell lymphoma, glioma growth factor), and IL-6 (growth factor for multiple myeloma), thereby promoting the above signaling pathways regulated by VEGF and IL-6. Two small-molecule inhibitors regulate cell signaling pathways in a synergistic manner to inhibit the proliferation and migration of Tregs, thereby blocking the secretion of inhibitory cytokines by Tregs to aid in the formation of the TME. Currently, four clinical trials have been recruited on the https://www.clinicaltrials.gov/, and they involve solid tumors and leukemia.

## Conclusion

**FIGURE 3:**
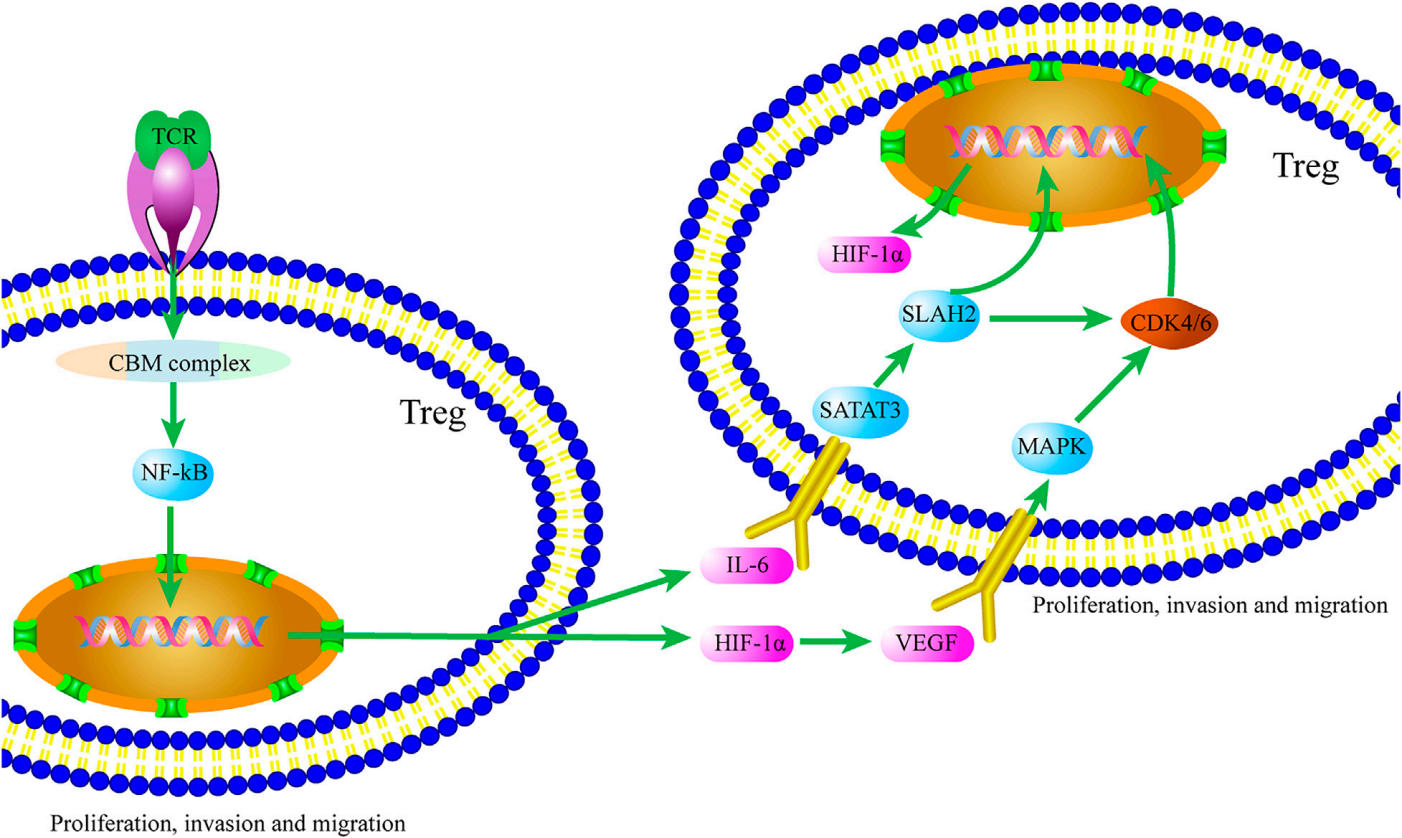
Crosstalk of the CBM complex/NF-κB and MAPK/P27 signaling pathways Tregs contributes to the TME. Ligand binding to TCR of Tregs, HIF-1α and IL-6 are produced via activating the CBM complex/NF-κB signaling pathway. In turn, Treg proliferation and migration are promoted by HIF-1α and IL-6 through the MAPK/CDK4/6/Rb and STAT3/SIAH2/P27 signaling pathways, respectively.

In conclusion, Tregs are involved in the regulation of autoimmune diseases, allergic diseases, and graft rejection, and Treg-mediated immunosuppression has become a major obstacle to effective treatment of tumors. Tregs play an immunosuppressive role in the TME through various mechanisms. Based on our analyses (Figure 3), we think that after specific ligand binding to TCR of Tregs, the CBM complex/NF-κB signaling pathway is activated, and factors such as HIF-1α and IL-6 are produced, thereby initiating the TME formation. HIF-1α and IL-6 promote Treg proliferation and migration through the MAPK/CDK4/6/Rb and STAT3/SIAH2/P27 signaling pathways, respectively. IL-6 also promotes the production of HIF-1α and enhances the self-regulation of Tregs in the process of TME formation.

In this review, we discussed the crosstalk between the CBM and MAPK/P27 signaling pathways, in order to gain a better understanding of the complexity of the role of Tregs in the process of TME formation. However, the complex role of Tregs in the formation of the TME *via* either CBM or the MAPK/P27 signaling pathway and its underlying mechanisms need further exploration. Therefore, further exploration of the complex role of CBM and MAPK/P27 signaling pathway in the formation of TME will provide stronger evidence for potential therapeutic targets in solid tumor therapy.
